# Supplementary material for: From Young to Older, the 4 Phases Method Is Efficient in Promoting Quick Weight, BMI, and Waist Circumference Reductions
Source: Healthcare (Basel). 2022 Jul 27;10(8):1398. doi: 10.3390/healthcare10081398 (PMC9332815; doi:10.3390/healthcare10081398)
Supplement: Supplementary file 1 [file healthcare-10-01398-s001.zip › healthcare-1815304-supplementary.pdf]

Supplementary Material: Participant's data - weight, BMI, and waist circumference reductions (%) after 5 weeks of 4 Phases Methodology.

SEX	AGE	WEIGHT BASELINE (KG)	WEIGHT POST (KG)	WEIGHT LOSS (KG)	WEIGHT LOSS (%)	HEIGHT (M)	BMI BASELINE	BMI POST	BMI REDUCTION	BMI REDUCTION (%)	WC BASELINE (CM)	WC POST (CM)	WC REDUCTION (CM)	WC REDUCTION (%)
F	46,00	71,00	60,00	-11,00	*-15,49%*	1,65	26,08	22,04	-4,04	*-15,49%*	90,00	81,00	-9,00	*-10,00%*
F	46,00	78,00	71,00	-7,00	*-8,97%*	1,62	29,72	27,05	-2,67	*-8,97%*	97,00	91,00	-6,00	*-6,19%*
F	34,00	79,90	71,30	-8,60	*-10,76%*	1,59	31,60	28,20	-3,40	*-10,76%*	85,00	79,00	-6,00	*-7,06%*
F	62,00	90,70	83,70	-7,00	*-7,72%*	1,63	34,14	31,50	-2,63	*-7,72%*	109,00	103,00	-6,00	*-5,50%*
F	52,00	88,50	81,90	-6,60	*-7,46%*	1,53	37,81	34,99	-2,82	*-7,46%*	109,00	102,00	-7,00	*-6,42%*
F	25,00	90,40	84,00	-6,40	*-7,08%*	1,60	35,31	32,81	-2,50	*-7,08%*	107,00	101,00	-6,00	*-5,61%*
F	36,00	68,90	62,70	-6,20	*-9,00%*	1,61	26,58	24,19	-2,39	*-9,00%*	80,00	74,00	-6,00	*-7,50%*
F	33,00	98,00	91,90	-6,10	*-6,22%*	1,70	33,91	31,80	-2,11	*-6,22%*	96,00	92,00	-4,00	*-4,17%*
F	42,00	85,40	79,70	-5,70	*-6,67%*	1,63	32,14	30,00	-2,15	*-6,67%*	104,00	97,00	-7,00	*-6,73%*

SEX	AGE	WEIGHT BASELINE (KG)	WEIGHT POST (KG)	WEIGHT LOSS (KG)	WEIGHT LOSS (%)	HEIGHT (M)	BMI BASELINE	BMI POST	BMI REDUCTION	BMI REDUCTION (%)	WC BASELINE (CM)	WC POST (CM)	WC REDUCTION (CM)	WC REDUCTION (%)
F	41,00	78,10	72,80	-5,30	*-6,79%*	1,65	28,69	26,74	-1,95	*-6,79%*	93,00	89,00	-4,00	*-4,30%*
F	41,00	68,60	63,00	-5,60	*-8,16%*	1,51	30,09	27,63	-2,46	*-8,16%*	87,00	80,00	-7,00	*-8,05%*
F	22,00	77,30	72,30	-5,00	*-6,47%*	1,64	28,74	26,88	-1,86	*-6,47%*	95,00	85,00	-10,00	*-10,53%*
F	23,00	70,70	68,50	-2,20	*-3,11%*	1,55	29,43	28,51	-0,92	*-3,11%*	92,00	92,00	0,00	*0,00%*
F	28,00	66,10	64,80	-1,30	*-1,97%*	1,62	25,19	24,69	-0,50	*-1,97%*	86,00	83,00	-3,00	*-3,49%*
F	36,00	89,00	86,00	-3,00	*-3,37%*	1,58	35,65	34,45	-1,20	*-3,37%*	104,00	98,00	-6,00	*-5,77%*
F	44,00	105,40	101,30	-4,10	*-3,89%*	1,65	38,71	37,21	-1,51	*-3,89%*	117,00	107,00	-10,00	*-8,55%*
F	38,00	116,30	110,50	-5,80	*-4,99%*	1,78	36,71	34,88	-1,83	*-4,99%*	119,00	116,00	-3,00	*-2,52%*
F	39,00	82,40	76,10	-6,30	*-7,65%*	1,50	36,62	33,82	-2,80	*-7,65%*	96,00	93,00	-3,00	*-3,13%*
F	46,00	87,00	73,80	-13,20	*-15,17%*	1,60	33,98	28,83	-5,16	*-15,17%*	103,00	83,00	-20,00	*-19,42%*
F	50,00	68,50	60,00	-8,50	*-12,41%*	1,53	29,26	25,63	-3,63	*-12,41%*	83,00	77,00	-6,00	*-7,23%*
F	39,00	121,30	114,20	-7,10	*-5,85%*	1,80	37,44	35,25	-2,19	*-5,85%*	119,00	111,00	-8,00	*-6,72%*
F	59,00	103,40	96,40	-7,00	*-6,77%*	1,56	42,49	39,61	-2,88	*-6,77%*	107,00	97,00	-10,00	*-9,35%*
F	67,00	106,70	99,20	-7,50	*-7,03%*	1,62	40,66	37,80	-2,86	*-7,03%*	107,00	93,00	-14,00	*-13,08%*
F	51,00	82,50	75,50	-7,00	*-8,48%*	1,64	30,67	28,07	-2,60	*-8,48%*	98,00	93,00	-5,00	*-5,10%*
F	43,00	120,00	109,70	-10,30	*-8,58%*	1,60	46,88	42,85	-4,02	*-8,58%*	112,00	100,00	-12,00	*-10,71%*
F	28,00	96,60	87,90	-8,70	*-9,01%*	1,59	38,21	34,77	-3,44	*-9,01%*	108,00	101,00	-7,00	*-6,48%*

F	18,00	129,00	120,70	-8,30	*-6,43%*	1,69	45,17	42,26	-2,91	*-6,43%*	124,00	118,00	-6,00	*-4,84%*
F	54,00	124,70	109,10	-15,60	*-12,51%*	1,58	49,95	43,70	-6,25	*-12,51%*	124,00	114,00	-10,00	*-8,06%*
F	29,00	86,50	76,00	-10,50	*-12,14%*	1,62	32,96	28,96	-4,00	*-12,14%*	92,00	84,00	-8,00	*-8,70%*
F	35,00	95,30	85,90	-9,40	*-9,86%*	1,66	34,58	31,17	-3,41	*-9,86%*	105,00	96,00	-9,00	*-8,57%*
F	40,00	65,10	58,30	-6,80	*-10,45%*	1,54	27,45	24,58	-2,87	*-10,45%*	95,00	88,00	-7,00	*-7,37%*
F	41,00	113,50	106,90	-6,60	*-5,81%*	1,55	47,24	44,50	-2,75	*-5,81%*	114,00	109,00	-5,00	*-4,39%*
F	34,00	72,10	66,80	-5,30	*-7,35%*	1,67	25,85	23,95	-1,90	*-7,35%*	87,00	81,00	-6,00	*-6,90%*
F	50,00	114,70	104,20	-10,50	*-9,15%*	1,65	42,13	38,27	-3,86	*-9,15%*	111,00	103,00	-8,00	*-7,21%*
F	47,00	100,20	94,60	-5,60	*-5,59%*	1,70	34,67	32,73	-1,94	*-5,59%*	117,00	110,00	-7,00	*-5,98%*
F	37,00	91,70	83,70	-8,00	*-8,72%*	1,65	33,68	30,74	-2,94	*-8,72%*	95,00	86,00	-9,00	*-9,47%*
F	40,00	114,50	107,20	-7,30	*-6,38%*	1,68	40,57	37,98	-2,59	*-6,38%*	118,00	111,00	-7,00	*-5,93%*
F	64,00	119,20	110,90	-8,30	*-6,96%*	1,55	49,61	46,16	-3,45	*-6,96%*	117,00	108,00	-9,00	*-7,69%*
F	50,00	79,80	73,10	-6,70	*-8,40%*	1,62	30,41	27,85	-2,55	*-8,40%*	97,00	85,00	-12,00	*-12,37%*
F	22,00	82,50	75,00	-7,50	*-9,09%*	1,61	31,83	28,93	-2,89	*-9,09%*	85,00	79,00	-6,00	*-7,06%*
F	41,00	87,70	81,30	-6,40	*-7,30%*	1,59	34,69	32,16	-2,53	*-7,30%*	102,00	96,00	-6,00	*-5,88%*
F	40,00	106,10	96,80	-9,30	*-8,77%*	1,69	37,15	33,89	-3,26	*-8,77%*	103,00	95,50	-7,50	*-7,28%*

F	51,00	89,00	87,70	-1,30	*-1,46%*	1,59	35,20	34,69	-0,51	*-1,46%*	113,00	105,00	-8,00	*-7,08%*
F	27,00	84,60	76,80	-7,80	*-9,22%*	1,56	34,76	31,56	-3,21	*-9,22%*	105,00	96,00	-9,00	*-8,57%*
F	27,00	88,20	82,10	-6,10	*-6,92%*	1,59	34,89	32,47	-2,41	*-6,92%*	97,00	93,00	-4,00	*-4,12%*
F	40,00	82,00	71,70	-10,30	*-12,56%*	1,53	35,03	30,63	-4,40	*-12,56%*	105,00	97,50	-7,50	*-7,14%*
F	34,00	92,60	85,50	-7,10	*-7,67%*	1,56	38,05	35,13	-2,92	*-7,67%*	115,00	108,00	-7,00	*-6,09%*
F	26,00	89,70	83,00	-6,70	*-7,47%*	1,60	35,04	32,42	-2,62	*-7,47%*	95,00	90,00	-5,00	*-5,26%*
F	45,00	82,50	75,40	-7,10	*-8,61%*	1,52	35,71	32,64	-3,07	*-8,61%*	105,00	99,00	-6,00	*-5,71%*
F	37,00	135,50	129,20	-6,30	*-4,65%*	1,74	44,75	42,67	-2,08	*-4,65%*	123,00	119,00	-4,00	*-3,25%*
F	51,00	97,10	90,50	-6,60	*-6,80%*	1,56	39,90	37,19	-2,71	*-6,80%*	117,00	113,00	-4,00	*-3,42%*
F	46,00	69,70	64,60	-5,10	*-7,32%*	1,50	30,98	28,71	-2,27	*-7,32%*	112,00	108,00	-4,00	*-3,57%*
F	33,00	85,80	74,80	-11,00	*-12,82%*	1,65	31,52	27,47	-4,04	*-12,82%*	96,00	94,00	-2,00	*-2,08%*
F	48,00	74,80	69,50	-5,30	*-7,09%*	1,59	29,59	27,49	-2,10	*-7,09%*	89,50	87,00	-2,50	*-2,79%*
F	29,00	78,50	73,00	-5,50	*-7,01%*	1,57	31,85	29,62	-2,23	*-7,01%*	95,00	91,00	-4,00	*-4,21%*
F	36,00	100,00	91,90	-8,10	*-8,10%*	1,63	37,64	34,59	-3,05	*-8,10%*	105,00	101,50	-3,50	*-3,33%*
F	42,00	90,80	83,00	-7,80	*-8,59%*	1,62	34,60	31,63	-2,97	*-8,59%*	113,00	106,00	-7,00	*-6,19%*
F	25,00	69,00	63,00	-6,00	*-8,70%*	1,58	27,64	25,24	-2,40	*-8,70%*	95,00	77,00	-18,00	*-18,95%*

F	40,00	98,00	86,40	-11,60	*-11,84%*	1,68	34,72	30,61	-4,11	*-11,84%*	118,00	105,00	-13,00	*-11,02%*
F	29,00	97,50	87,00	-10,50	*-10,77%*	1,63	36,70	32,74	-3,95	*-10,77%*	105,00	99,00	-6,00	*-5,71%*
F	34,00	119,40	101,70	-17,70	*-14,82%*	1,70	41,31	35,19	-6,12	*-14,82%*	114,00	107,00	-7,00	*-6,14%*
F	30,00	90,20	80,80	-9,40	*-10,42%*	1,60	35,23	31,56	-3,67	*-10,42%*	95,00	86,00	-9,00	*-9,47%*
F	23,00	69,20	61,20	-8,00	*-11,56%*	1,56	28,44	25,15	-3,29	*-11,56%*	93,00	81,00	-12,00	*-12,90%*
F	40,00	77,10	67,00	-10,10	*-13,10%*	1,60	30,12	26,17	-3,95	*-13,10%*	95,00	88,00	-7,00	*-7,37%*
F	46,00	96,40	87,40	-9,00	*-9,34%*	1,63	36,28	32,90	-3,39	*-9,34%*	116,00	96,00	-20,00	*-17,24%*
F	52,00	109,50	99,60	-9,90	*-9,04%*	1,68	38,80	35,29	-3,51	*-9,04%*	122,00	116,00	-6,00	*-4,92%*
F	41,00	100,40	89,60	-10,80	*-10,76%*	1,64	37,33	33,31	-4,02	*-10,76%*	117,00	100,00	-17,00	*-14,53%*
F	45,00	83,60	72,10	-11,50	*-13,76%*	1,63	31,47	27,14	-4,33	*-13,76%*	100,00	84,00	-16,00	*-16,00%*
F	36,00	113,70	103,40	-10,30	*-9,06%*	1,60	44,41	40,39	-4,02	*-9,06%*	120,00	110,00	-10,00	*-8,33%*
F	22,00	120,90	111,70	-9,20	*-7,61%*	1,71	41,35	38,20	-3,15	*-7,61%*	106,00	96,00	-10,00	*-9,43%*
F	46,00	86,20	78,50	-7,70	*-8,93%*	1,57	34,97	31,85	-3,12	*-8,93%*	96,00	87,00	-9,00	*-9,38%*
F	36,00	102,60	95,30	-7,30	*-7,12%*	1,60	40,08	37,23	-2,85	*-7,12%*	124,00	119,00	-5,00	*-4,03%*
F	58,00	107,60	100,50	-7,10	*-6,60%*	1,60	42,03	39,26	-2,77	*-6,60%*	112,00	106,00	-6,00	*-5,36%*
F	43,00	101,50	94,60	-6,90	*-6,80%*	1,71	34,71	32,35	-2,36	*-6,80%*	115,00	107,00	-8,00	*-6,96%*

F	27,00	120,20	113,80	-6,40	*-5,32%*	1,60	46,95	44,45	-2,50	*-5,32%*	137,00	126,00	-11,00	*-8,03%*
F	44,00	101,50	93,20	-8,30	*-8,18%*	1,76	32,77	30,09	-2,68	*-8,18%*	105,00	97,00	-8,00	*-7,62%*
F	28,00	101,10	93,70	-7,40	*-7,32%*	1,55	42,08	39,00	-3,08	*-7,32%*	104,00	96,00	-8,00	*-7,69%*
F	42,00	82,20	75,90	-6,30	*-7,66%*	1,59	32,51	30,02	-2,49	*-7,66%*	107,00	95,00	-12,00	*-11,21%*
F	24,00	77,30	71,50	-5,80	*-7,50%*	1,65	28,39	26,26	-2,13	*-7,50%*	96,00	84,00	-12,00	*-12,50%*
F	52,00	77,20	71,60	-5,60	*-7,25%*	1,68	27,35	25,37	-1,98	*-7,25%*	98,00	85,00	-13,00	*-13,27%*
F	51,00	82,50	77,10	-5,40	*-6,55%*	1,56	33,90	31,68	-2,22	*-6,55%*	122,00	101,00	-21,00	*-17,21%*
F	58,00	70,50	65,20	-5,30	*-7,52%*	1,52	30,51	28,22	-2,29	*-7,52%*	93,00	86,00	-7,00	*-7,53%*
F	52,00	80,40	76,10	-4,30	*-5,35%*	1,55	33,47	31,68	-1,79	*-5,35%*	102,00	96,00	-6,00	*-5,88%*
F	36,00	75,00	63,20	-11,80	*-15,73%*	1,60	29,30	24,69	-4,61	*-15,73%*	102,00	86,00	-16,00	*-15,69%*
F	26,00	122,40	112,40	-10,00	*-8,17%*	1,69	42,86	39,35	-3,50	*-8,17%*	134,00	126,00	-8,00	*-5,97%*
F	53,00	90,20	79,70	-10,50	*-11,64%*	1,70	31,21	27,58	-3,63	*-11,64%*	108,00	90,00	-18,00	*-16,67%*
F	32,00	95,60	83,50	-12,10	*-12,66%*	1,67	34,28	29,94	-4,34	*-12,66%*	111,00	92,00	-19,00	*-17,12%*
F	66,00	114,10	102,00	-12,10	*-10,60%*	1,72	38,57	34,48	-4,09	*-10,60%*	118,00	105,00	-13,00	*-11,02%*
F	28,00	101,10	86,00	-15,10	*-14,94%*	1,75	33,01	28,08	-4,93	*-14,94%*	136,00	105,00	-31,00	*-22,79%*
F	29,00	82,20	70,10	-12,10	*-14,72%*	1,63	30,94	26,38	-4,55	*-14,72%*	104,00	84,00	-20,00	*-19,23%*

F	37,00	82,00	76,30	-5,70	*-6,95%*	1,67	29,40	27,36	-2,04	*-6,95%*	109,00	103,00	-6,00	*-5,50%*
F	25,00	97,90	88,40	-9,50	*-9,70%*	1,58	39,22	35,41	-3,81	*-9,70%*	120,00	115,00	-5,00	*-4,17%*
F	40,00	137,30	124,40	-12,90	*-9,40%*	1,76	44,32	40,16	-4,16	*-9,40%*	136,00	127,00	-9,00	*-6,62%*
F	31,00	68,90	64,20	-4,70	*-6,82%*	1,64	25,62	23,87	-1,75	*-6,82%*	90,00	84,00	-6,00	*-6,67%*
F	24,00	113,60	104,80	-8,80	*-7,75%*	1,70	39,31	36,26	-3,04	*-7,75%*	111,00	109,50	-1,50	*-1,35%*
F	61,00	65,40	55,10	-10,30	*-15,75%*	1,52	28,31	23,85	-4,46	*-15,75%*	91,00	76,00	-15,00	*-16,48%*
F	27,00	105,90	97,50	-8,40	*-7,93%*	1,68	37,52	34,55	-2,98	*-7,93%*	115,00	111,00	-4,00	*-3,48%*
F	37,00	72,70	67,10	-5,60	*-7,70%*	1,70	25,16	23,22	-1,94	*-7,70%*	98,00	94,00	-4,00	*-4,08%*
F	38,00	73,00	67,30	-5,70	*-7,81%*	1,50	32,44	29,91	-2,53	*-7,81%*	105,00	96,00	-9,00	*-8,57%*
F	39,00	86,70	78,60	-8,10	*-9,34%*	1,56	35,63	32,30	-3,33	*-9,34%*	114,00	106,00	-8,00	*-7,02%*
F	35,00	129,80	114,10	-15,70	*-12,10%*	1,72	43,88	38,57	-5,31	*-12,10%*	131,50	121,50	-10,00	*-7,60%*
F	38,00	78,70	71,50	-7,20	*-9,15%*	1,71	26,91	24,45	-2,46	*-9,15%*	95,00	85,00	-10,00	*-10,53%*
F	36,00	73,80	66,60	-7,20	*-9,76%*	1,63	27,78	25,07	-2,71	*-9,76%*	88,00	83,00	-5,00	*-5,68%*
F	29,00	77,20	70,60	-6,60	*-8,55%*	1,65	28,36	25,93	-2,42	*-8,55%*	84,50	75,30	-9,20	*-10,89%*
F	35,00	65,10	59,50	-5,60	*-8,60%*	1,61	25,11	22,95	-2,16	*-8,60%*	77,50	71,50	-6,00	*-7,74%*
F	53,00	68,90	63,40	-5,50	*-7,98%*	1,76	22,24	20,47	-1,78	*-7,98%*	91,50	86,00	-5,50	*-6,01%*

F	21,00	82,10	74,70	-7,40	-9,01%	1,85	23,99	21,83	-2,16	-9,01%	92,00	86,00	-6,00	-6,52%
F	24,00	77,90	71,20	-6,70	-8,60%	1,69	27,27	24,93	-2,35	-8,60%	86,00	81,00	-5,00	-5,81%
F	30,00	74,40	68,40	-6,00	-8,06%	1,70	25,74	23,67	-2,08	-8,06%	89,00	81,00	-8,00	-8,99%
F	45,00	69,20	60,40	-8,80	-12,72%	1,58	27,72	24,19	-3,53	-12,72%	105,00	96,00	-9,00	-8,57%
F	42,00	68,50	62,50	-6,00	-8,76%	1,63	25,78	23,52	-2,26	-8,76%	93,20	90,00	-3,20	-3,43%
F	63,00	84,70	77,70	-7,00	-8,26%	1,59	33,50	30,73	-2,77	-8,26%	122,00	112,00	-10,00	-8,20%
F	42,00	91,40	84,10	-7,30	-7,99%	1,67	32,77	30,16	-2,62	-7,99%	106,00	100,00	-6,00	-5,66%
F	39,00	117,50	109,60	-7,90	-6,72%	1,78	37,08	34,59	-2,49	-6,72%	131,00	128,50	-2,50	-1,91%
F	23,00	84,30	74,40	-9,90	-11,74%	1,57	34,20	30,18	-4,02	-11,74%	98,00	90,00	-8,00	-8,16%
F	46,00	90,00	81,80	-8,20	-9,11%	1,62	34,29	31,17	-3,12	-9,11%	91,00	89,00	-2,00	-2,20%
F	58,00	98,00	89,80	-8,20	-8,37%	1,58	39,26	35,97	-3,28	-8,37%	110,00	108,50	-1,50	-1,36%
F	45,00	77,50	71,90	-5,60	-7,23%	1,65	28,47	26,41	-2,06	-7,23%	81,00	76,00	-5,00	-6,17%
F	54,00	89,00	81,00	-8,00	-8,99%	1,61	34,34	31,25	-3,09	-8,99%	100,00	91,00	-9,00	-9,00%
F	20,00	80,30	70,60	-9,70	-12,08%	1,63	30,22	26,57	-3,65	-12,08%	90,00	80,00	-10,00	-11,11%
F	52,00	100,70	90,50	-10,20	-10,13%	1,69	35,26	31,69	-3,57	-10,13%	111,00	100,00	-11,00	-9,91%
F	40,00	80,30	74,50	-5,80	-7,22%	1,62	30,60	28,39	-2,21	-7,22%	89,00	82,00	-7,00	-7,87%

F	45,00	111,30	105,10	-6,20	*-5,57%*	1,78	35,13	33,17	-1,96	*-5,57%*	110,00	104,00	-6,00	*-5,45%*
F	32,00	99,30	90,80	-8,50	*-8,56%*	1,68	35,18	32,17	-3,01	*-8,56%*	105,00	91,00	-14,00	*-13,33%*
F	39,00	113,30	106,80	-6,50	*-5,74%*	1,73	37,86	35,68	-2,17	*-5,74%*	115,00	115,00	0,00	*0,00%*
F	48,00	63,70	56,50	-7,20	*-11,30%*	1,60	24,88	22,07	-2,81	*-11,30%*	77,00	73,00	-4,00	*-5,19%*
F	39,00	82,30	75,30	-7,00	*-8,51%*	1,58	32,97	30,16	-2,80	*-8,51%*	99,00	87,00	-12,00	*-12,12%*
F	42,00	71,20	65,60	-5,60	*-7,87%*	1,56	29,26	26,96	-2,30	*-7,87%*	92,00	81,00	-11,00	*-11,96%*
F	50,00	126,20	116,70	-9,50	*-7,53%*	1,85	36,87	34,10	-2,78	*-7,53%*	124,00	119,00	-5,00	*-4,03%*
F	36,00	115,60	106,90	-8,70	*-7,53%*	1,78	36,49	33,74	-2,75	*-7,53%*	118,00	112,00	-6,00	*-5,08%*
F	30,00	118,60	109,90	-8,70	*-7,34%*	1,76	38,29	35,48	-2,81	*-7,34%*	120,00	112,00	-8,00	*-6,67%*
F	18,00	117,10	106,60	-10,50	*-8,97%*	1,80	36,14	32,90	-3,24	*-8,97%*	120,00	109,00	-11,00	*-9,17%*
F	48,00	137,70	127,70	-10,00	*-7,26%*	1,80	42,50	39,41	-3,09	*-7,26%*	127,00	114,00	-13,00	*-10,24%*
F	36,00	94,40	84,60	-9,80	*-10,38%*	1,63	35,53	31,84	-3,69	*-10,38%*	102,00	95,00	-7,00	*-6,86%*
F	19,00	74,10	71,10	-3,00	*-4,05%*	1,74	24,47	23,48	-0,99	*-4,05%*	81,00	76,00	-5,00	*-6,17%*
F	37,00	85,10	77,90	-7,20	*-8,46%*	1,88	24,08	22,04	-2,04	*-8,46%*	99,00	89,00	-10,00	*-10,10%*
F	23,00	88,50	82,30	-6,20	*-7,01%*	1,61	34,14	31,75	-2,39	*-7,01%*	108,00	99,00	-9,00	*-8,33%*
F	36,00	97,70	94,40	-3,30	*-3,38%*	1,69	34,21	33,05	-1,16	*-3,38%*	109,00	105,00	-4,00	*-3,67%*

F	30,00	88,00	80,80	-7,20	*-8,18%*	1,63	33,12	30,41	-2,71	*-8,18%*	97,00	89,00	-8,00	*-8,25%*
F	21,00	98,40	90,30	-8,10	*-8,23%*	1,70	34,05	31,25	-2,80	*-8,23%*	99,00	92,00	-7,00	*-7,07%*
F	49,00	61,40	56,43	-4,97	*-8,09%*	1,72	20,75	19,07	-1,68	*-8,09%*	83,50	78,50	-5,00	*-5,99%*
F	36,00	80,00	72,60	-7,40	*-9,25%*	1,75	26,12	23,71	-2,42	*-9,25%*	99,00	93,00	-6,00	*-6,06%*
F	21,00	61,00	56,00	-5,00	*-8,20%*	1,70	21,11	19,38	-1,73	*-8,20%*	85,00	78,00	-7,00	*-8,24%*
F	38,00	75,60	69,60	-6,00	*-7,94%*	1,60	29,53	27,19	-2,34	*-7,94%*	90,00	85,00	-5,00	*-5,56%*
F	49,00	124,10	108,30	-15,80	*-12,73%*	1,76	40,06	34,96	-5,10	*-12,73%*	124,00	117,00	-7,00	*-5,65%*
F	45,00	84,90	78,00	-6,90	*-8,13%*	1,66	30,81	28,31	-2,50	*-8,13%*	109,00	99,00	-10,00	*-9,17%*
F	28,00	60,70	55,50	-5,20	*-8,57%*	1,60	23,71	21,68	-2,03	*-8,57%*	83,00	79,00	-4,00	*-4,82%*
F	35,00	88,80	82,70	-6,10	*-6,87%*	1,75	29,00	27,00	-1,99	*-6,87%*	99,50	94,50	-5,00	*-5,03%*
F	31,00	95,70	89,50	-6,20	*-6,48%*	1,73	31,98	29,90	-2,07	*-6,48%*	108,50	103,50	-5,00	*-4,61%*
F	28,00	92,40	86,60	-5,80	*-6,28%*	1,74	30,52	28,60	-1,92	*-6,28%*	111,00	105,00	-6,00	*-5,41%*
F	29,00	76,20	73,10	-3,10	*-4,07%*	1,70	26,37	25,29	-1,07	*-4,07%*	99,50	96,00	-3,50	*-3,52%*
F	47,00	76,00	69,00	-7,00	*-9,21%*	1,53	32,47	29,48	-2,99	*-9,21%*	99,00	95,00	-4,00	*-4,04%*
F	42,00	86,00	78,00	-8,00	*-9,30%*	1,63	32,37	29,36	-3,01	*-9,30%*	101,50	96,50	-5,00	*-4,93%*
F	18,00	97,30	89,00	-8,30	*-8,53%*	1,71	33,28	30,44	-2,84	*-8,53%*	98,00	88,00	-10,00	*-10,20%*

F	41,00	81,30	73,10	-8,20	*-10,09%*	1,59	32,16	28,91	-3,24	*-10,09%*	88,00	82,00	-6,00	*-6,82%*
F	56,00	79,10	73,90	-5,20	*-6,57%*	1,79	24,69	23,06	-1,62	*-6,57%*	92,00	87,00	-5,00	*-5,43%*
F	36,00	66,10	60,00	-6,10	*-9,23%*	1,53	28,24	25,63	-2,61	*-9,23%*	83,00	76,00	-7,00	*-8,43%*
F	28,00	77,10	72,70	-4,40	*-5,71%*	1,60	30,12	28,40	-1,72	*-5,71%*	88,00	83,00	-5,00	*-5,68%*
F	41,00	90,60	85,60	-5,00	*-5,52%*	1,69	31,72	29,97	-1,75	*-5,52%*	88,00	84,00	-4,00	*-4,55%*
F	36,00	83,60	75,30	-8,30	*-9,93%*	1,56	34,35	30,94	-3,41	*-9,93%*	99,00	92,00	-7,00	*-7,07%*
F	25,00	71,20	64,40	-6,80	*-9,55%*	1,59	28,16	25,47	-2,69	*-9,55%*	95,00	87,00	-8,00	*-8,42%*
F	22,00	124,20	111,90	-12,30	*-9,90%*	1,83	37,09	33,41	-3,67	*-9,90%*	127,00	116,00	-11,00	*-8,66%*
F	35,00	165,50	154,20	-11,30	*-6,83%*	1,90	45,84	42,71	-3,13	*-6,83%*	155,00	148,00	-7,00	*-4,52%*
F	18,00	89,00	82,80	-6,20	*-6,97%*	1,63	33,50	31,16	-2,33	*-6,97%*	105,00	99,00	-6,00	*-5,71%*
F	42,00	164,40	154,40	-10,00	*-6,08%*	1,82	49,63	46,61	-3,02	*-6,08%*	136,00	128,00	-8,00	*-5,88%*
F	49,00	83,60	78,00	-5,60	*-6,70%*	1,62	31,85	29,72	-2,13	*-6,70%*	103,00	98,00	-5,00	*-4,85%*
F	43,00	55,70	47,30	-8,40	*-15,08%*	1,52	24,11	20,47	-3,64	*-15,08%*	86,00	80,00	-6,00	*-6,98%*
F	22,00	84,10	79,00	-5,10	*-6,06%*	1,51	36,88	34,65	-2,24	*-6,06%*	100,00	99,00	-1,00	*-1,00%*
F	44,00	61,90	54,20	-7,70	*-12,44%*	1,50	27,51	24,09	-3,42	*-12,44%*	93,00	84,00	-9,00	*-9,68%*
F	51,00	75,60	70,60	-5,00	*-6,61%*	1,66	27,44	25,62	-1,81	*-6,61%*	93,00	88,00	-5,00	*-5,38%*

F	37,00	62,30	57,10	-5,20	*-8,35%*	1,76	20,11	18,43	-1,68	*-8,35%*	89,00	84,00	-5,00	*-5,62%*
F	48,00	80,90	73,90	-7,00	*-8,65%*	1,72	27,35	24,98	-2,37	*-8,65%*	102,00	98,00	-4,00	*-3,92%*
F	44,00	139,90	127,70	-12,20	*-8,72%*	1,61	53,97	49,27	-4,71	*-8,72%*	136,00	120,00	-16,00	*-11,76%*
F	39,00	95,00	86,30	-8,70	*-9,16%*	1,73	31,74	28,83	-2,91	*-9,16%*	107,00	96,00	-11,00	*-10,28%*
F	52,00	73,50	65,50	-8,00	*-10,88%*	1,55	30,59	27,26	-3,33	*-10,88%*	93,00	88,00	-5,00	*-5,38%*
F	46,00	111,00	104,10	-6,90	*-6,22%*	1,76	35,83	33,61	-2,23	*-6,22%*	115,00	109,00	-6,00	*-5,22%*
F	39,00	90,50	84,20	-6,30	*-6,96%*	1,60	35,35	32,89	-2,46	*-6,96%*	100,00	98,00	-2,00	*-2,00%*
F	47,00	74,20	68,10	-6,10	*-8,22%*	1,63	27,93	25,63	-2,30	*-8,22%*	90,00	81,00	-9,00	*-10,00%*
F	44,00	89,80	83,80	-6,00	*-6,68%*	1,67	32,20	30,05	-2,15	*-6,68%*	120,00	116,00	-4,00	*-3,33%*
F	43,00	105,10	99,10	-6,00	*-5,71%*	1,68	37,24	35,11	-2,13	*-5,71%*	114,00	107,00	-7,00	*-6,14%*
F	30,00	72,40	66,80	-5,60	*-7,73%*	1,72	24,47	22,58	-1,89	*-7,73%*	89,00	82,00	-7,00	*-7,87%*
F	43,00	108,70	98,40	-10,30	*-9,48%*	1,70	37,61	34,05	-3,56	*-9,48%*	110,00	105,00	-5,00	*-4,55%*
F	23,00	87,40	80,20	-7,20	*-8,24%*	1,55	36,38	33,38	-3,00	*-8,24%*	106,00	100,00	-6,00	*-5,66%*
F	29,00	99,20	93,10	-6,10	*-6,15%*	1,71	33,92	31,84	-2,09	*-6,15%*	104,00	100,00	-4,00	*-3,85%*
F	27,00	91,40	83,70	-7,70	*-8,42%*	1,60	35,70	32,70	-3,01	*-8,42%*	101,00	90,00	-11,00	*-10,89%*
F	39,00	62,50	57,70	-4,80	*-7,68%*	1,57	25,36	23,41	-1,95	*-7,68%*	81,00	71,00	-10,00	*-12,35%*

F	30,00	94,90	85,60	-9,30	*-9,80%*	1,85	27,73	25,01	-2,72	*-9,80%*	104,00	94,00	-10,00	*-9,62%*
F	34,00	85,20	77,20	-8,00	*-9,39%*	1,70	29,48	26,71	-2,77	*-9,39%*	96,00	89,00	-7,00	*-7,29%*
F	41,00	120,50	114,50	-6,00	*-4,98%*	1,74	39,80	37,82	-1,98	*-4,98%*	127,00	123,00	-4,00	*-3,15%*
F	43,00	67,60	61,40	-6,20	*-9,17%*	1,64	25,13	22,83	-2,31	*-9,17%*	77,00	74,00	-3,00	*-3,90%*
F	49,00	105,40	97,60	-7,80	*-7,40%*	1,68	37,34	34,58	-2,76	*-7,40%*	120,00	116,00	-4,00	*-3,33%*
F	65,00	93,20	88,20	-5,00	*-5,36%*	1,63	35,08	33,20	-1,88	*-5,36%*	107,00	103,00	-4,00	*-3,74%*
F	42,00	80,10	75,20	-4,90	*-6,12%*	1,67	28,72	26,96	-1,76	*-6,12%*	83,00	78,00	-5,00	*-6,02%*
F	34,00	93,40	86,10	-7,30	*-7,82%*	1,74	30,85	28,44	-2,41	*-7,82%*	100,00	91,00	-9,00	*-9,00%*
F	35,00	124,30	111,90	-12,40	*-9,98%*	1,88	35,17	31,66	-3,51	*-9,98%*	124,00	110,00	-14,00	*-11,29%*
F	39,00	62,40	55,30	-7,10	*-11,38%*	1,61	24,07	21,33	-2,74	*-11,38%*	91,00	78,00	-13,00	*-14,29%*
F	32,00	77,20	73,20	-4,00	*-5,18%*	1,67	27,68	26,25	-1,43	*-5,18%*	83,00	79,00	-4,00	*-4,82%*
F	28,00	60,50	52,60	-7,90	*-13,06%*	1,63	22,77	19,80	-2,97	*-13,06%*	77,00	69,00	-8,00	*-10,39%*
F	64,00	78,10	71,40	-6,70	*-8,58%*	1,63	29,40	26,87	-2,52	*-8,58%*	100,00	91,00	-9,00	*-9,00%*
F	38,00	91,40	83,90	-7,50	*-8,21%*	1,70	31,63	29,03	-2,60	*-8,21%*	105,00	97,00	-8,00	*-7,62%*
F	34,00	114,90	106,40	-8,50	*-7,40%*	1,72	38,84	35,97	-2,87	*-7,40%*	113,00	102,00	-11,00	*-9,73%*
F	30,00	72,40	66,80	-5,60	*-7,73%*	1,74	23,91	22,06	-1,85	*-7,73%*	89,00	82,00	-7,00	*-7,87%*

F	53,00	128,70	118,40	-10,30	*-8,00%*	1,52	55,70	51,25	-4,46	*-8,00%*	120,00	115,00	-5,00	*-4,17%*
F	23,00	87,40	80,20	-7,20	*-8,24%*	1,73	29,20	26,80	-2,41	*-8,24%*	106,00	100,00	-6,00	*-5,66%*
F	29,00	99,20	93,10	-6,10	*-6,15%*	1,79	30,96	29,06	-1,90	*-6,15%*	104,00	100,00	-4,00	*-3,85%*
F	27,00	91,40	83,70	-7,70	*-8,42%*	1,69	32,00	29,31	-2,70	*-8,42%*	109,00	98,00	-11,00	*-10,09%*
F	39,00	62,50	57,70	-4,80	*-7,68%*	1,58	25,04	23,11	-1,92	*-7,68%*	81,00	71,00	-10,00	*-12,35%*
F	34,00	71,00	60,00	-11,00	*-15,49%*	1,65	26,08	22,04	-4,04	*-15,49%*	90,00	81,00	-9,00	*-10,00%*
F	44,00	78,00	71,00	-7,00	*-8,97%*	1,62	29,72	27,05	-2,67	*-8,97%*	97,00	91,00	-6,00	*-6,19%*
F	46,00	89,90	81,30	-8,60	*-9,57%*	1,59	35,56	32,16	-3,40	*-9,57%*	85,00	79,00	-6,00	*-7,06%*
F	61,00	93,70	86,70	-7,00	*-7,47%*	1,64	34,84	32,24	-2,60	*-7,47%*	109,00	103,00	-6,00	*-5,50%*
F	51,00	78,50	80,90	2,40	*3,06%*	1,61	30,28	31,21	0,93	*3,06%*	109,00	102,00	-7,00	*-6,42%*
F	25,00	91,40	85,00	-6,40	*-7,00%*	1,70	31,63	29,41	-2,21	*-7,00%*	97,00	91,00	-6,00	*-6,19%*
F	24,00	70,90	64,70	-6,20	*-8,74%*	1,61	27,35	24,96	-2,39	*-8,74%*	90,00	84,00	-6,00	*-6,67%*
F	32,00	98,00	91,90	-6,10	*-6,22%*	1,67	35,14	32,95	-2,19	*-6,22%*	96,00	92,00	-4,00	*-4,17%*
F	29,00	84,40	78,70	-5,70	*-6,75%*	1,63	31,77	29,62	-2,15	*-6,75%*	104,00	97,00	-7,00	*-6,73%*
F	40,00	78,10	72,80	-5,30	*-6,79%*	1,65	28,69	26,74	-1,95	*-6,79%*	83,00	79,00	-4,00	*-4,82%*
F	42,00	69,60	64,00	-5,60	*-8,05%*	1,85	20,34	18,70	-1,64	*-8,05%*	87,00	80,00	-7,00	*-8,05%*

F	24,00	76,30	71,30	-5,00	*-6,55%*	1,64	28,37	26,51	-1,86	*-6,55%*	95,00	85,00	-10,00	*-10,53%*
F	22,00	70,70	68,50	-2,20	*-3,11%*	1,55	29,43	28,51	-0,92	*-3,11%*	92,00	92,00	0,00	*0,00%*
F	28,00	65,10	63,80	-1,30	*-2,00%*	1,62	24,81	24,31	-0,50	*-2,00%*	86,00	83,00	-3,00	*-3,49%*
F	36,00	109,00	106,00	-3,00	*-2,75%*	1,74	36,00	35,01	-0,99	*-2,75%*	104,00	98,00	-6,00	*-5,77%*
F	39,00	114,40	110,30	-4,10	*-3,58%*	1,76	36,93	35,61	-1,32	*-3,58%*	117,00	107,00	-10,00	*-8,55%*
F	36,00	124,30	118,50	-5,80	*-4,67%*	1,81	37,94	36,17	-1,77	*-4,67%*	119,00	116,00	-3,00	*-2,52%*
F	40,00	82,40	76,10	-6,30	*-7,65%*	1,71	28,18	26,03	-2,15	*-7,65%*	96,00	93,00	-3,00	*-3,13%*
F	42,00	85,00	71,80	-13,20	*-15,53%*	1,60	33,20	28,05	-5,16	*-15,53%*	103,00	83,00	-20,00	*-19,42%*
F	52,00	129,50	121,00	-8,50	*-6,56%*	1,78	40,87	38,19	-2,68	*-6,56%*	123,00	117,00	-6,00	*-4,88%*
F	38,00	120,30	113,20	-7,10	*-5,90%*	1,71	41,14	38,71	-2,43	*-5,90%*	119,00	111,00	-8,00	*-6,72%*
F	59,00	94,40	87,40	-7,00	*-7,42%*	1,68	33,45	30,97	-2,48	*-7,42%*	107,00	97,00	-10,00	*-9,35%*
F	65,00	104,70	97,20	-7,50	*-7,16%*	1,62	39,89	37,04	-2,86	*-7,16%*	107,00	93,00	-14,00	*-13,08%*
F	52,00	83,50	76,50	-7,00	*-8,38%*	1,64	31,05	28,44	-2,60	*-8,38%*	98,00	93,00	-5,00	*-5,10%*
F	45,00	100,20	89,40	-10,80	*-10,78%*	1,60	39,14	34,92	-4,22	*-10,78%*	99,00	90,00	-9,00	*-9,09%*
F	27,00	106,90	97,30	-9,60	*-8,98%*	1,73	35,72	32,51	-3,21	*-8,98%*	108,00	101,00	-7,00	*-6,48%*
F	19,00	110,00	101,70	-8,30	*-7,55%*	1,69	38,51	35,61	-2,91	*-7,55%*	124,00	118,00	-6,00	*-4,84%*

F	52,00	104,20	89,10	-15,10	*-14,49%*	1,83	31,11	26,61	-4,51	*-14,49%*	124,00	114,00	-10,00	*-8,06%*
F	24,00	87,50	77,00	-10,50	*-12,00%*	1,62	33,34	29,34	-4,00	*-12,00%*	92,00	84,00	-8,00	*-8,70%*
F	25,00	85,30	75,90	-9,40	*-11,02%*	1,66	30,96	27,54	-3,41	*-11,02%*	105,00	96,00	-9,00	*-8,57%*
F	35,00	65,90	58,80	-7,10	*-10,77%*	1,84	19,46	17,37	-2,10	*-10,77%*	95,00	88,00	-7,00	*-7,37%*
F	41,00	122,50	115,90	-6,60	*-5,39%*	1,83	36,58	34,61	-1,97	*-5,39%*	114,00	109,00	-5,00	*-4,39%*
F	35,00	72,70	66,20	-6,50	*-8,94%*	1,67	26,07	23,74	-2,33	*-8,94%*	87,00	81,00	-6,00	*-6,90%*
F	52,00	116,10	105,80	-10,30	*-8,87%*	1,72	39,24	35,76	-3,48	*-8,87%*	120,00	112,00	-8,00	*-6,67%*
F	46,00	103,20	97,60	-5,60	*-5,43%*	1,70	35,71	33,77	-1,94	*-5,43%*	107,00	100,00	-7,00	*-6,54%*
F	33,00	91,20	83,10	-8,10	*-8,88%*	1,65	33,50	30,52	-2,98	*-8,88%*	99,00	90,00	-9,00	*-9,09%*
F	42,00	135,50	128,20	-7,30	*-5,39%*	1,68	48,01	45,42	-2,59	*-5,39%*	118,00	111,00	-7,00	*-5,93%*
F	59,00	129,90	121,70	-8,20	*-6,31%*	1,70	44,95	42,11	-2,84	*-6,31%*	127,00	118,00	-9,00	*-7,09%*
F	51,00	77,80	71,10	-6,70	*-8,61%*	1,62	29,64	27,09	-2,55	*-8,61%*	97,00	85,00	-12,00	*-12,37%*
F	22,00	85,50	78,40	-7,10	*-8,30%*	1,61	32,98	30,25	-2,74	*-8,30%*	90,00	84,00	-6,00	*-6,67%*
F	43,00	107,20	101,50	-5,70	*-5,32%*	1,67	38,44	36,39	-2,04	*-5,32%*	112,00	106,00	-6,00	*-5,36%*
F	39,00	114,10	104,80	-9,30	*-8,15%*	1,69	39,95	36,69	-3,26	*-8,15%*	103,00	95,50	-7,50	*-7,28%*
F	48,00	89,90	87,20	-2,70	*-3,00%*	1,73	30,04	29,14	-0,90	*-3,00%*	113,00	105,00	-8,00	*-7,08%*

F	24,00	84,10	76,30	-7,80	*-9,27%*	1,74	27,78	25,20	-2,58	*-9,27%*	105,00	96,00	-9,00	*-8,57%*
F	27,00	90,20	84,90	-5,30	*-5,88%*	1,59	35,68	33,58	-2,10	*-5,88%*	97,00	93,00	-4,00	*-4,12%*
F	38,00	102,50	91,90	-10,60	*-10,34%*	1,67	36,75	32,95	-3,80	*-10,34%*	105,00	97,50	-7,50	*-7,14%*
F	33,00	92,60	82,50	-10,10	*-10,91%*	1,89	25,92	23,10	-2,83	*-10,91%*	115,00	108,00	-7,00	*-6,09%*
F	26,00	91,30	85,40	-5,90	*-6,46%*	1,68	32,35	30,26	-2,09	*-6,46%*	95,00	90,00	-5,00	*-5,26%*
F	39,00	85,30	78,80	-6,50	*-7,62%*	1,52	36,92	34,11	-2,81	*-7,62%*	105,00	99,00	-6,00	*-5,71%*
F	36,00	125,50	119,50	-6,00	*-4,78%*	1,79	39,17	37,30	-1,87	*-4,78%*	123,00	119,00	-4,00	*-3,25%*
F	43,00	107,90	100,80	-7,10	*-6,58%*	1,75	35,23	32,91	-2,32	*-6,58%*	117,00	113,00	-4,00	*-3,42%*
F	52,00	117,10	112,60	-4,50	*-3,84%*	1,78	36,96	35,54	-1,42	*-3,84%*	117,00	113,00	-4,00	*-3,42%*
F	34,00	85,20	74,90	-10,30	*-12,09%*	1,65	31,29	27,51	-3,78	*-12,09%*	96,00	94,00	-2,00	*-2,08%*
M	28,00	97,00	92,00	-5,00	*-5,15%*	1,89	27,15	25,76	-1,39	*-5,12%*	96,00	93,00	-3,00	*-3,13%*
M	16,00	87,20	78,90	-8,30	*-9,52%*	1,73	29,14	26,36	-2,78	*-9,54%*	94,00	88,00	-6,00	*-6,38%*
M	38,00	96,50	88,60	-7,90	*-8,19%*	1,65	35,45	32,54	-2,91	*-8,21%*	109,00	102,00	-7,00	*-6,42%*
M	30,00	170,00	151,50	-18,50	*-10,88%*	1,62	64,78	57,73	-7,05	*-10,88%*	145,00	138,00	-7,00	*-4,83%*
M	34,00	114,20	103,70	-10,50	*-9,19%*	1,72	38,60	35,05	-3,55	*-9,20%*	122,00	117,00	-5,00	*-4,10%*
M	30,00	88,60	79,20	-9,40	*-10,61%*	1,78	27,96	25,00	-2,96	*-10,59%*	109,00	99,00	-10,00	*-9,17%*

M	34,00	105,00	96,30	-8,70	*-8,29%*	1,84	31,01	28,44	-2,57	*-8,29%*	116,00	107,00	-9,00	*-7,76%*
M	49,00	123,20	114,90	-8,30	*-6,74%*	1,80	38,02	35,46	-2,56	*-6,73%*	126,00	124,00	-2,00	*-1,59%*
M	27,00	106,70	98,40	-8,30	*-7,78%*	1,80	32,93	30,37	-2,56	*-7,77%*	109,00	103,00	-6,00	*-5,50%*
M	46,00	100,90	93,70	-7,20	*-7,14%*	1,78	31,85	29,57	-2,28	*-7,16%*	114,00	108,00	-6,00	*-5,26%*
M	33,00	80,90	70,30	-10,60	*-13,10%*	1,73	27,03	23,49	-3,54	*-13,10%*	95,00	82,00	-13,00	*-13,68%*
M	33,00	113,90	104,50	-9,40	*-8,25%*	1,75	37,19	34,12	-3,07	*-8,25%*	114,00	109,00	-5,00	*-4,39%*
M	40,00	123,00	114,20	-8,80	*-7,15%*	1,78	38,82	36,04	-2,78	*-7,16%*	120,00	113,00	-7,00	*-5,83%*
M	30,00	73,10	67,00	-6,10	*-8,34%*	1,60	28,55	26,17	-2,38	*-8,34%*	87,00	82,00	-5,00	*-5,75%*
M	44,00	115,80	107,00	-8,80	*-7,60%*	1,87	33,12	30,60	-2,52	*-7,61%*	113,00	104,00	-9,00	*-7,96%*
M	51,00	112,00	105,20	-6,80	*-6,07%*	1,79	34,96	32,83	-2,13	*-6,09%*	118,00	110,00	-8,00	*-6,78%*
M	36,00	77,00	70,80	-6,20	*-8,05%*	1,68	27,28	25,09	-2,19	*-8,03%*	100,00	94,00	-6,00	*-6,00%*
M	28,00	108,20	101,10	-7,10	*-6,56%*	1,75	35,33	33,01	-2,32	*-6,57%*	117,00	107,00	-10,00	*-8,55%*
M	35,00	102,10	96,00	-6,10	*-5,97%*	1,76	32,96	30,99	-1,97	*-5,98%*	104,00	99,00	-5,00	*-4,81%*
M	42,00	121,10	106,50	-14,60	*-12,06%*	1,80	37,38	32,87	-4,51	*-12,07%*	133,00	122,00	-11,00	*-8,27%*
M	26,00	146,90	138,20	-8,70	*-5,92%*	1,82	44,35	41,72	-2,63	*-5,93%*	127,00	122,00	-5,00	*-3,94%*
M	31,00	136,80	128,70	-8,10	*-5,92%*	1,96	35,61	33,50	-2,11	*-5,93%*	127,00	118,00	-9,00	*-7,09%*

M	18,00	130,50	121,90	-8,60	*-6,59%*	1,86	37,72	35,24	-2,48	*-6,57%*	121,00	116,00	-5,00	*-4,13%*
M	34,00	91,60	85,90	-5,70	*-6,22%*	1,77	29,24	27,42	-1,82	*-6,22%*	101,00	94,00	-7,00	*-6,93%*
M	35,00	127,00	112,30	-14,70	*-11,57%*	1,89	35,55	31,44	-4,11	*-11,56%*	122,00	101,00	-21,00	*-17,21%*
M	58,00	118,10	101,40	-16,70	*-14,14%*	1,75	38,56	33,11	-5,45	*-14,13%*	136,00	117,00	-19,00	*-13,97%*
M	35,00	98,30	80,40	-17,90	*-18,21%*	1,72	33,23	27,18	-6,05	*-18,21%*	112,00	94,00	-18,00	*-16,07%*
M	29,00	100,80	90,30	-10,50	*-10,42%*	1,80	31,11	27,87	-3,24	*-10,41%*	104,00	96,50	-7,50	*-7,21%*
M	51,00	140,00	131,00	-9,00	*-6,43%*	1,80	43,21	40,43	-2,78	*-6,43%*	140,00	131,00	-9,00	*-6,43%*
M	40,00	137,30	124,40	-12,90	*-9,40%*	1,80	42,38	38,40	-3,98	*-9,39%*	136,00	127,00	-9,00	*-6,62%*
M	30,00	180,10	166,00	-14,10	*-7,83%*	1,78	56,84	52,39	-4,45	*-7,83%*	158,00	153,00	-5,00	*-3,16%*
M	31,00	91,40	83,00	-8,40	*-9,19%*	1,77	29,17	26,49	-2,68	*-9,19%*	97,00	89,00	-8,00	*-8,25%*
M	45,00	128,00	117,00	-11,00	*-8,59%*	1,79	39,95	36,52	-3,43	*-8,59%*	133,00	122,00	-11,00	*-8,27%*
M	26,00	78,30	71,00	-7,30	*-9,32%*	1,74	25,86	23,45	-2,41	*-9,32%*	101,00	93,00	-8,00	*-7,92%*
M	27,00	92,40	83,90	-8,50	*-9,20%*	1,77	29,49	26,78	-2,71	*-9,19%*	106,00	94,00	-12,00	*-11,32%*
M	54,00	78,00	71,40	-6,60	*-8,46%*	1,75	25,47	23,31	-2,16	*-8,48%*	101,00	92,00	-9,00	*-8,91%*
M	41,00	134,00	121,30	-12,70	*-9,48%*	1,91	36,73	33,25	-3,48	*-9,47%*	129,00	119,00	-10,00	*-7,75%*
M	31,00	89,40	80,60	-8,80	*-9,84%*	1,74	29,53	26,62	-2,91	*-9,85%*	102,00	94,00	-8,00	*-7,84%*

M	32,00	96,70	90,75	-5,95	*-6,15%*	1,75	31,58	29,63	-1,95	*-6,17%*	111,00	104,50	-6,50	*-5,86%*
M	30,00	88,00	77,90	-10,10	*-11,48%*	1,74	29,07	25,73	-3,34	*-11,49%*	110,00	100,00	-10,00	*-9,09%*
M	35,00	87,20	78,50	-8,70	*-9,98%*	1,75	28,47	25,63	-2,84	*-9,98%*	100,00	98,00	-2,00	*-2,00%*
M	37,00	99,80	93,10	-6,70	*-6,71%*	1,70	34,53	32,21	-2,32	*-6,72%*	111,00	105,00	-6,00	*-5,41%*
M	20,00	119,70	114,80	-4,90	*-4,09%*	1,73	39,99	38,36	-1,63	*-4,08%*	125,00	117,00	-8,00	*-6,40%*
M	49,00	90,90	83,70	-7,20	*-7,92%*	1,55	37,84	34,84	-3,00	*-7,93%*	105,00	96,00	-9,00	*-8,57%*
M	41,00	92,40	85,40	-7,00	*-7,58%*	1,58	37,01	34,21	-2,80	*-7,57%*	111,00	105,00	-6,00	*-5,41%*
M	37,00	121,70	113,30	-8,40	*-6,90%*	1,77	38,85	36,16	-2,69	*-6,92%*	121,00	117,00	-4,00	*-3,31%*
M	27,00	108,30	98,30	-10,00	*-9,23%*	1,61	41,78	37,92	-3,86	*-9,24%*	129,00	120,00	-9,00	*-6,98%*
M	21,00	103,70	93,40	-10,30	*-9,93%*	1,77	33,10	29,81	-3,29	*-9,94%*	110,00	106,00	-4,00	*-3,64%*
M	42,00	82,50	77,20	-5,30	*-6,42%*	1,53	35,24	32,98	-2,26	*-6,41%*	105,00	95,00	-10,00	*-9,52%*
M	42,00	72,40	67,40	-5,00	*-6,91%*	1,67	25,96	24,17	-1,79	*-6,90%*	91,00	87,00	-4,00	*-4,40%*
M	52,00	77,60	72,20	-5,40	*-6,96%*	1,70	26,85	24,98	-1,87	*-6,96%*	97,00	94,20	-2,80	*-2,89%*
M	44,00	71,30	66,80	-4,50	*-6,31%*	1,48	32,55	30,50	-2,05	*-6,30%*	98,00	93,00	-5,00	*-5,10%*
M	48,00	86,00	81,50	-4,50	*-5,23%*	1,70	29,76	28,20	-1,56	*-5,24%*	100,00	95,00	-5,00	*-5,00%*
M	35,00	94,50	88,50	-6,00	*-6,35%*	1,70	32,70	30,62	-2,08	*-6,36%*	105,00	105,00	0,00	*0,00%*

M	43,00	78,20	73,50	-4,70	*-6,01%*	1,66	28,38	26,67	-1,71	*-6,03%*	99,00	93,50	-5,50	*-5,56%*
M	38,00	98,50	89,20	-9,30	*-9,44%*	1,65	36,18	32,76	-3,42	*-9,45%*	117,50	109,00	-8,50	*-7,23%*
M	38,00	108,00	97,90	-10,10	*-9,35%*	1,73	36,09	32,71	-3,38	*-9,37%*	108,00	102,00	-6,00	*-5,56%*
M	57,00	76,30	70,20	-6,10	*-7,99%*	1,59	30,18	27,77	-2,41	*-7,99%*	104,00	95,00	-9,00	*-8,65%*
M	41,00	88,00	83,00	-5,00	*-5,68%*	1,56	36,16	34,11	-2,05	*-5,67%*	95,00	92,00	-3,00	*-3,16%*
M	26,00	96,60	91,00	-5,60	*-5,80%*	1,74	31,91	30,06	-1,85	*-5,80%*	103,00	96,00	-7,00	*-6,80%*
M	43,00	83,80	77,70	-6,10	*-7,28%*	1,70	29,00	26,89	-2,11	*-7,28%*	104,00	97,00	-7,00	*-6,73%*
M	49,00	88,80	84,20	-4,60	*-5,18%*	1,72	30,02	28,46	-1,55	*-5,18%*	112,00	106,00	-6,00	*-5,36%*
M	47,00	74,50	66,30	-8,20	*-11,01%*	1,68	26,40	23,49	-2,91	*-11,02%*	97,00	88,00	-9,00	*-9,28%*
M	46,00	117,50	108,00	-9,50	*-8,09%*	1,77	37,51	34,47	-3,04	*-8,10%*	124,00	120,00	-4,00	*-3,23%*
M	46,00	79,00	74,50	-4,50	*-5,70%*	1,59	31,25	29,47	-1,78	*-5,70%*	97,00	95,00	-2,00	*-2,06%*
M	39,00	97,60	88,50	-9,10	*-9,32%*	1,51	42,81	38,81	-4,00	*-9,34%*	115,00	103,00	-12,00	*-10,43%*
M	24,00	115,50	105,80	-9,70	*-8,40%*	1,78	36,45	33,39	-3,06	*-8,40%*	117,00	111,00	-6,00	*-5,13%*
M	38,00	87,20	82,00	-5,20	*-5,96%*	1,77	27,83	26,17	-1,66	*-5,96%*	99,00	93,00	-6,00	*-6,06%*
M	27,00	99,00	89,10	-9,90	*-10,00%*	1,65	36,36	32,73	-3,63	*-9,98%*	105,00	98,00	-7,00	*-6,67%*
M	25,00	88,30	81,30	-7,00	*-7,93%*	1,68	31,29	28,81	-2,48	*-7,93%*	92,00	87,00	-5,00	*-5,43%*

M	67,00	56,90	51,50	-5,40	*-9,49%*	1,58	22,79	20,63	-2,16	*-9,48%*	86,00	82,00	-4,00	*-4,65%*
M	41,00	97,30	90,90	-6,40	*-6,58%*	1,78	30,71	28,69	-2,02	*-6,58%*	108,00	103,00	-5,00	*-4,63%*
M	33,00	105,60	95,10	-10,50	*-9,94%*	1,90	29,25	26,34	-2,91	*-9,95%*	117,00	113,00	-4,00	*-3,42%*
M	64,00	66,80	61,60	-5,20	*-7,78%*	1,52	28,91	26,66	-2,25	*-7,78%*	93,00	80,00	-13,00	*-13,98%*
M	37,00	77,80	68,40	-9,40	*-12,08%*	1,68	27,57	24,23	-3,34	*-12,11%*	96,00	85,00	-11,00	*-11,46%*
M	39,00	76,50	70,40	-6,10	*-7,97%*	1,62	29,15	26,83	-2,32	*-7,96%*	87,00	82,00	-5,00	*-5,75%*
M	24,00	93,90	86,60	-7,30	*-7,77%*	1,65	34,49	31,81	-2,68	*-7,77%*	100,00	97,00	-3,00	*-3,00%*
M	23,00	95,40	88,50	-6,90	*-7,23%*	1,80	29,44	27,31	-2,13	*-7,24%*	108,00	93,00	-15,00	*-13,89%*
M	52,00	91,50	84,00	-7,50	*-8,20%*	1,53	39,09	35,88	-3,21	*-8,21%*	107,00	96,00	-11,00	*-10,28%*
M	38,00	75,70	70,00	-5,70	*-7,53%*	1,60	29,57	27,34	-2,23	*-7,54%*	98,00	94,00	-4,00	*-4,08%*
M	67,00	56,90	51,50	-5,40	*-9,49%*	1,62	21,68	19,62	-2,06	*-9,50%*	86,00	82,00	-4,00	*-4,65%*
M	41,00	98,30	91,90	-6,40	*-6,51%*	1,75	31,77	29,68	-2,09	*-6,58%*	118,00	113,00	-5,00	*-4,24%*
M	33,00	125,60	115,10	-10,50	*-8,36%*	1,80	32,59	29,35	-3,24	*-9,94%*	127,00	123,00	-4,00	*-3,15%*
M	30,00	94,90	85,60	-9,30	*-9,80%*	1,82	28,65	25,84	-2,81	*-9,81%*	104,00	94,00	-10,00	*-9,62%*
M	36,00	78,90	72,70	-6,20	*-7,86%*	1,61	30,44	28,05	-2,39	*-7,86%*	88,00	82,00	-6,00	*-6,82%*
M	33,00	88,00	81,90	-6,10	*-6,93%*	1,67	31,55	29,37	-2,19	*-6,93%*	94,00	90,00	-4,00	*-4,26%*

M	39,00	121,30	114,20	-7,10	*-5,85%*	1,58	44,58	41,74	-2,84	*-6,38%*	124,00	116,00	-8,00	*-6,45%*
M	34,00	96,20	88,20	-8,00	*-8,32%*	1,70	32,94	30,17	-2,77	*-8,40%*	106,00	99,00	-7,00	*-6,60%*
M	36,00	93,60	85,30	-8,30	*-8,87%*	1,56	38,46	35,05	-3,41	*-8,87%*	98,00	91,00	-7,00	*-7,14%*
M	35,00	114,30	101,90	-12,40	*-10,85%*	1,88	32,34	28,83	-3,51	*-10,85%*	125,00	111,00	-14,00	*-11,20%*
M	41,00	130,50	124,50	-6,00	*-4,60%*	1,74	43,10	41,12	-1,98	*-4,60%*	129,00	125,00	-4,00	*-3,10%*
M	28,00	70,50	62,60	-7,90	*-11,21%*	1,63	26,53	23,56	-2,97	*-11,21%*	78,00	70,00	-8,00	*-10,26%*
M	30,00	83,40	77,80	-5,60	*-6,71%*	1,72	27,85	25,96	-1,89	*-6,80%*	98,00	91,00	-7,00	*-7,14%*
M	43,00	77,60	71,40	-6,20	*-7,99%*	1,64	28,85	26,55	-2,31	*-7,99%*	78,00	75,00	-3,00	*-3,85%*

**Mean of weight reduction after 5 weeks of 4 Phases Method**	*-8.8% (- 7.6 Kg)*

**Mean of waist circumference reduction after 5 weeks of 4 Phases Method**	*-7.1% (- 7.4 cm)*

**Mean of BMI reduction after 5 weeks of 4 Phases Method**	*-8.8%*
